# EuGMS Task and Finish group on Fall-Risk-Increasing Drugs (FRIDs): Position on Knowledge Dissemination, Management, and Future Research

**DOI:** 10.1007/s40266-018-0622-7

**Published:** 2019-02-11

**Authors:** L. J. Seppala, N. van der Velde, T. Masud, H. Blain, M. Petrovic, T. J. van der Cammen, K. Szczerbińska, S. Hartikainen, R. A. Kenny, J. Ryg, P. Eklund, E. Topinková, A. Mair, L. Laflamme, H. Thaler, G. Bahat, M. Gutiérrez-Valencia, MA Caballero-Mora, F. Landi, M. H. Emmelot-Vonk, the EuGMS Task and Finish Group on Fall-Risk-Increasing Drugs, A. Cherubini, J. P. Baeyens, A. Correa-Pérez, A. Gudmundsson, A. Marengoni, D. O’Mahony, N. Parekh, F. E. Pisa, C. Rajkumar, M. Wehling, G. Ziere, the EuGMS Special Interest Group on Pharmacology

**Affiliations:** 1 Department of Internal Medicine, Section of Geriatric Medicine, Amsterdam Public Health Research Institute, Amsterdam UMC, University of Amsterdam, Meibergdreef 9, 1105AZ Amsterdam, The Netherlands; 2 Nottingham University Hospitals NHS Trust, Nottingham, UK; 3 Department of Internal Medicine and Geriatrics, University Hospital of Montpellier, Montpellier University, Euromov, France; 4 Department of Internal Medicine (Geriatrics), Ghent University, Ghent, Belgium; 5 Faculty of Industrial Design Engineering, Delft University of Technology, Delft, The Netherlands; 6 Unit for Research on Aging Society, Department of Sociology of Medicine, Epidemiology and Preventive Medicine Chair, Faculty of Medicine, Jagiellonian University Medical College, Krakow, Poland; 7 School of Pharmacy, University of Eastern Finland, Kuopio, Finland; 8 The Irish Longitudinal Study on Ageing (TILDA), Trinity College Dublin, Dublin 2, Ireland; 9 Department of Medical Gerontology, School of Medicine, Trinity College Dublin, Dublin 2, Ireland; 10 Falls and Syncope Unit, Mercer’s Institute for Successful Ageing, St. James’s Hospital, Dublin 8, Ireland; 11 Department of Geriatric Medicine, Odense University Hospital, Odense, Denmark; 12 Geriatric Research Unit, Department of Clinical Research, University of Southern Denmark, Odense, Denmark; 13 Department of Computing Science, Umeå University, Umeå, Sweden; 14 Department of Geriatrics and Gerontology, 1st Faculty of Medicine, Charles University, Prague, Czech Republic; 15 Faculty of Health and Social Sciences, South Bohemian University, Česke Budějovice, Czech Republic; 16 Effective Prescribing and Therapeutics, Health and Social Care Directorate, Scottish Government, Edinburgh, Scotland, UK; 17 Department of Public Health Sciences, Karolinska Institutet, Tomtebodavägen 18A, Widerströmska huset, 17177 Stockholm, Sweden; 18 Trauma Center Wien-Meidling, Kundratstrasse 37, 1120 Vienna, Austria; 19 Istanbul Medical School, Department of Internal Medicine, Division of Geriatrics, Istanbul University, Capa, 34093 Istanbul, Turkey; 20 Department of Health Sciences, Public University of Navarra (UPNA), Avda, Barañain s/n, 31008 Pamplona, Spain; 21 Servicio de Geriatría, Hospital Universitario de Getafe and CIBER de Fragilidad y Envejecimiento Saludable, Getafe, Madrid Spain; 22 Department of Gerontology, Neuroscience and Orthopedics, Catholic University of the Sacred Heart, Rome, Italy; 23 Department of Geriatrics, University Medical Center Utrecht, Utrecht University, Utrecht, The Netherlands; 24 Geriatria, Accettazione geriatrica e Centro di ricerca per l’Invecchiamento, Italian National Research Center on Aging (INRCA), Ancona, Italy; 25 University of Luxembourg, Ezch-sur-Alzette, Luxembourg; 26 AZ Alma, Eeklo, Belgium; 27 Servicio de Geriatría, Hospital Universitario Ramón y Cajal (IRYCIS), Madrid, Spain; 28 Landspitali University Hospital, Reykjavik, Iceland; 29 Faculty of Medicine, University of Iceland, Reykjavik, Iceland; 30 Department of Clinical and Experimental Science, University of Brescia, Brescia, Italy; 31 Department of Geriatric Medicine, Cork University Hospital, Cork, Ireland; 32 Department of Medicine, University College Cork, Cork, Ireland; 33 Academic Department of Geriatric Medicine, Brighton and Sussex Medical School, Brighton, Sussex UK; 34 Department of Clinical Epidemiology, Leibniz Institute for Prevention Research and Epidemiology-BIPS, Bremen, Germany; 35 Institute of Hygiene and Clinical Epidemiology, University Hospital of Udine, Udine, Italy; 36 Department of Elderly Medicine, Brighton and Sussex University Hospitals NHS Trust, Sussex, UK; 37 Institute of Clinical Pharmacology Mannheim, University of Heidelberg, Heidelberg, Germany; 38 Department of Internal Medicine, Erasmus MC, University Medical Centre Rotterdam, Rotterdam, The Netherlands; 39 Department of Epidemiology, Erasmus MC, University Medical Centre Rotterdam, Rotterdam, The Netherlands

## Abstract

Falls are a major public health concern in the older population, and certain medication classes are a significant risk factor for falls. However, knowledge is lacking among both physicians and older people, including caregivers, concerning the role of medication as a risk factor. In the present statement, the European Geriatric Medicine Society (EuGMS) Task and Finish group on fall-risk-increasing drugs (FRIDs), in collaboration with the EuGMS Special Interest group on Pharmacology and the European Union of Medical Specialists (UEMS) Geriatric Medicine Section, outlines its position regarding knowledge dissemination on medication-related falls in older people across Europe. The EuGMS Task and Finish group is developing educational materials to facilitate knowledge dissemination for healthcare professionals and older people. In addition, steps in primary prevention through judicious prescribing, deprescribing of FRIDs (withdrawal and dose reduction), and gaps in current research are outlined in this position paper.

## Key Points

**Table:** 

Falls are under-recognized as adverse drug events.
Healthcare professionals are reluctant to withdraw fall-risk-increasing medications.
The EuGMS Task and Finish group on fall-risk-increasing drugs (FRIDs) proposes in this paper its recommendations on dissemination of knowledge about, management of, and future research on FRIDs.

## Introduction

Falls in older people are a significant public health priority because of their high prevalence, related injuries, reduced quality of life for fallers, and the associated economic burden [1]. One-third of people aged ≥65 years fall at least once yearly, and 20% of these falls lead to severe injuries [2]. Among older people, approximately 40,000 fatal falls are reported in the EU annually [3]. Between 0.85 and 1.50% of the total healthcare expenditure in Europe, North America, and Australia [4] are fall-related costs, meaning these injuries are among the 20 most expensive medical conditions among community-dwelling older people [5]. Medications are crucial risk factors for falls, and withdrawal of fall-risk-increasing drugs (FRIDs) is an effective intervention to prevent falls [6]. Moreover, falls, widely acknowledged as a geriatric syndrome, are also established adverse drug events (ADEs). It is estimated that ADEs lead to 8.6 million unplanned hospital admissions in Europe annually, and 50% of these are preventable [7]. A total of 70% of ADEs affect patients aged > 65 years taking five or more medicines [7].

Despite the evidence of the association between medications and falls, awareness of this topic is lacking among physicians and older people and their caregivers [8–11]. Indeed, physicians and patients commonly overestimate the benefits of medications and underestimate the potential harms [12]. Furthermore, many physicians perceive the uncertainty about the consequences of withdrawing FRIDs as challenging and uncomfortable [9]. Older patients also have concerns about deprescribing, such as fearing a relapse of their condition, and concerns about adverse drug withdrawal reactions [13, 14]. Consequently, limited knowledge and skills in FRIDs withdrawal and reluctance to withdraw mean avoidable injurious events and other adverse outcomes related to falls continue.

In this position statement, the European Geriatric Medicine Society (EuGMS) Task and Finish group on FRIDs, in collaboration with the EuGMS Special Interest Group on Pharmacology and the European Union of Medical Specialists (UEMS)-Geriatric Medicine Section, outline (1) the preferential strategies for knowledge dissemination to older people and professionals on FRIDs, (2) recommendations for the management of FRIDs, and (3) recommendations for future research on medication-related falls.

## Prevention of Medication-Related Falls

Psychotropic and cardiovascular medicines are the most important FRID classes. The recent systematic reviews and meta-analyses by the EuGMS Task and Finish group on FRIDs confirmed the association between psychotropics (antidepressants [selective serotonin reuptake inhibitors, tricyclic antidepressants], antipsychotics, benzodiazepines) and fall risk [15]. Moreover, consistent associations with falls were reported for loop diuretics, antiepileptics, opioids, and polypharmacy (four or more medications) [16, 17]. Digitalis, non-selective beta-blocking agents, antiarrhythmics, diuretics in general, antihypertensives, anticholinergics, non-steroidal anti-inflammatory drugs, analgesics, laxatives, long-term proton pump inhibitors, and antiplatelets are also possible FRIDs [16, 17]. These high-risk medications are widely prescribed for older people. Almost 90% of older adults with dizziness visiting their general practitioner use at least one FRID [18]. Approximately 60% of older adults admitted to hospital because of a fall use one or more FRID, and 36% use five or more medications [19].

Preventing fall incidents by identifying and reducing the use of risk-increasing medications is an essential and effective component of a multifactorial fall-risk-management approach. A Cochrane review in 2012 summarized the randomized controlled trials (RCTs) of interventions to reduce falls in community-dwelling older people [6]. Gillespie et al. reported that withdrawal of psychotropics is effective in reducing fall rates and that a prescribing-modification program for primary care physicians can reduce the risk of falling in older people [6]. In addition, applying the FORTA (Fit fOR The Aged) list, a positive–negative medication-optimization approach, was effective in reducing fall rates in hospitalized geriatric patients [20]. Furthermore, the implementation of STOPP/START (Screening Tool for Older People’s potentially inappropriate Prescriptions/Screening Tool to Alert doctors to Right Treatment) criteria significantly reduced the number of falls in a chronic care geriatric facility [21]. The 2010 American Geriatrics Society/British Geriatrics Society joint guideline [22] and the World Health Organization (WHO) Integrated Care for Older People guideline [23] recommend that individuals at high risk of falling, identified by screening, should be assessed for risk factors, including medication. The medication review should include an assessment of drug duplicates and drug–drug interactions. The American Geriatrics Society/British Geriatrics Society joint guideline further states that minimizing medications is an important component of the multifactorial intervention and that, if discontinuation of a FRID is not possible, dose reduction should be considered.

Moreover, about 20% of falls result in serious injuries, such as fragility fractures and intracranial bleeding, and death [2]. Drug-induced osteoporosis is a growing health problem, and many commonly prescribed medications contribute to significant bone loss and fractures [24]. In the overall risk assessment of drugs contributing to falls and their serious consequences, the list of drugs known to enhance skeletal fragility (e.g., glucocorticoids, antiepileptics, gonadotropin-releasing hormone agonists and aromatase inhibitors) or bleeding (e.g., anticoagulants and antiplatelet therapy) should also be taken into account [25, 26].

Taken together, the EuGMS Task and Finish group on FRIDs

1. advocates better recognition of the role of FRIDs in fall incidents and the importance of knowledge dissemination on this topic,
2. advocates better research quality in the future to gain improved insight on FRIDs and their effective and safe withdrawal measures,
3. recommends systematically checking for a history of falls and high risk of falling before prescribing FRIDs,
4. supports and encourages better implementation of the 2010 American Geriatrics Society/British Geriatrics Society joint guideline recommendations into practice, including medication review for all patients with an acute fall, recurrent falls in the past year, or problems with walking or balance,
5. supports the EuGMS Falls and Fracture Special Interest Group recommendation that healthcare professionals screen older people, at least annually, for risk of falling [27], suggesting, in addition, a medication review for all older people every year, and every 6 months if the older individual is frail or vulnerable.

## How to Reduce the Use of Fall-Risk-Increasing Drugs (FRIDs)?

The first step in reducing the harm caused by FRIDs is to prevent their inappropriate use in the older population. Tools for detection of inappropriate prescribing, such as the Medication Appropriateness Index [28–30], have been developed to support a physician’s clinical judgment. It should be noted that listing approaches such as START/STOPP or FORTA include aspects of fall risk reduction, as FRIDs are mostly highlighted as causative factors. Interventional trials have shown that these systematic drug-optimization strategies can reduce drug side effects [31, 32], including falls [20]. However, no single ideal tool exists currently, and the choice of tool may rely on the purpose of use and availability of data [30, 33]. Such a tool should be not only well-designed and comprehensive but also practical to be implemented in everyday clinical practice [30, 33].

**Fig. 1:**
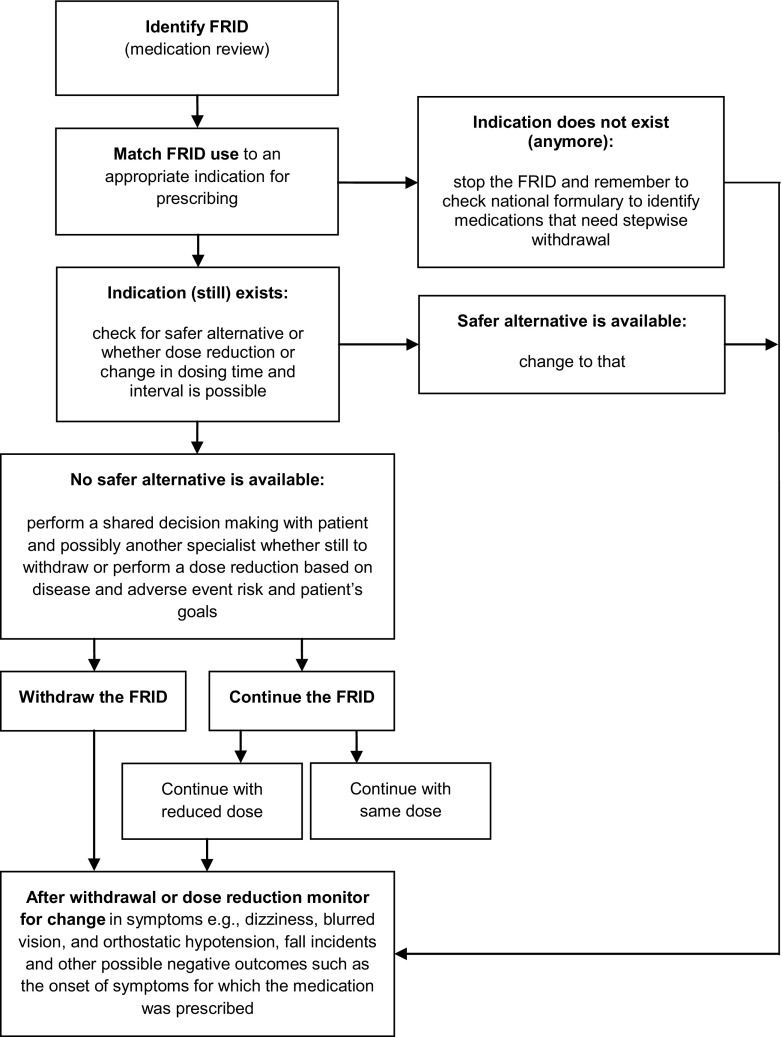
Decision tree for management of fall-risk-increasing drugs (FRIDs)

When reviewing a patient’s medication lists, withdrawal of FRIDs can be performed safely in older people at high risk of falls [34]. Withdrawal can involve immediate cessation of medication or a stepwise process depending on the medication to be withdrawn. For medications to be withdrawn in a stepwise manner, specific withdrawal guidelines are available in national formularies [35]. Examples of medications to be stopped gradually are benzodiazepines, opioids, antidepressants, and beta-blockers. The EuGMS Task and Finish group on FRIDs proposes the decision tree shown in Fig. 1 for withdrawal of FRIDs [36, 37].

## Multidisciplinary Approach

Healthcare professionals other than physicians, such as pharmacists, nurses, physiotherapists, occupational therapists, and nutritionists, play an important role in a multidisciplinary approach to prevention of drug-related falls. The American STEADI (Stopping Elderly Accidents, Deaths & Injuries) initiative concludes that a pharmacist can screen patients to determine fall risk using a standardized protocol, perform a medication review, and recommend vitamin D supplementation when appropriate [38]. Pharmacist-led medication improvement programs have led to improved medication use and greater discontinuation of high-risk medications [39, 40]. The pharmacist can arrange a fall risk assessment with a primary care team if the patient is at risk of falling, as identified through screening. In a thorough medication review, the pharmacist, in close collaboration with the multidisciplinary team and particularly the physician, can screen for FRIDs, assess the patient’s pharmacotherapy management, formulate the patient’s medication management plan, and educate the patient about fall-prevention strategies and medication changes [38]. The EuGMS Task and Finish group on FRIDs supports the recommendation of the STEADI initiative to promote greater involvement of a clinical pharmacist in the medication review process [38]. Similarly, nurses are well positioned to recognize a change in an older patient’s risk of falling and communicate this to the other multiprofessional team members [41, 42]. Nurses can drive organizational change toward falls prevention through a team approach by considering planning, implementing, and evaluating a falls-prevention program that incorporates identification and minimization of FRIDs [41, 42].

## Barriers to Effective Medication Withdrawals

Although withdrawal of FRIDs has been reported to be effective in reducing fall rates [6], the majority of older people do not have their medication checked or changed after a recent fall. An American survey found that only 3.5% of older people had their medication changed after a fall [43]. Furthermore, an observational study in primary care reported that withdrawal of FRIDs was performed in only 11.7% of seniors with a clear indication (dizziness) for withdrawal [18].

Lack of knowledge and skills are among the major barriers to providers adopting and implementing effective fall-prevention approaches [44]. These skills are not sufficiently acquired during studies. Physician trainees perceive difficulties dealing with multimorbidity, atypical disease presentations, and polypharmacy [45]. All these components are relevant for the recognition of and possible preventive interventions for medication-related falls.

For older people, a lack of perception about fall risk is a major barrier to patient participation in fall-prevention programs [46]. Older people often have an inappropriately positive perception of the possible consequences of a fall [47]. Moreover, awareness of medication management as an important fall-prevention strategy is low among older adults [8]. In addition, older people often do not consider medications as a possible risk factor for falls and struggle to present their medication-related problem to the physician [9]. Medication use is usually perceived as an unavoidable necessity [48].

## Improving Medication Withdrawal: Disseminating Knowledge

To overcome the lack of knowledge and skills among physicians and the poor awareness among older adults, the EuGMS Task and Finish group on FRIDs makes the following recommendations.

1. The European Undergraduate Curriculum in Geriatric Medicine should be mandatory in the education of all medical students. The curriculum states that graduates should know the pathophysiology, diagnosis, assessment, management, and preventive strategies for falls [49]. In addition, the EuGMS Task and Finish group on FRIDs is developing an English-language educational video regarding medication-related falls, which will be available through the website of the EuGMS Task and Finish group on FRIDs.
2. Knowledge should be disseminated among practising physicians and other healthcare professionals through seminars, brochures, web pages, and apps; by enlisting opinion leaders to influence colleagues; and by conducting educational outreach visits [50]. The Task and Finish group is developing English-language e-learning material about FRIDs and falls. We encourage European countries to adopt and test the material, translating where necessary (Table 1).

**Table 1:** Steps in translating educational material

1. Check the EuGMS Task and Finish group on FRIDs webpage (http://www.eugms.org/research-cooperation/special-interest-groups/falls-and-fractures.html) to see whether the educational material is available in your language
2. If not available in your language: The recommended way of translating the material is to use two translators, then conduct a pilot test among older adults, correct the translation, test again with older adults and then perform a reverse translation to confirm that the final version is consistent with the original version
3. If you perform a translation, contact us so we may make the material publically available through the EuGMS website

*EuGMS* European Geriatric Medicine Society, *FRIDs* fall-risk-increasing drugs
3. Public awareness and knowledge should be enhanced through media attention, educational material for older people, brochures, posters, and web pages [50]. Improving the health literacy of older adults could be a valuable intervention to reduce the harms associated with FRIDs [51]. For older people, we are designing an English-language leaflet about FRIDs for public dissemination. We encourage European countries to adopt and test the material, translating where appropriate (Table 1).
4. A European FRIDs list should be created. The EuGMS Task and Finish group plans to develop a FRIDs list to be used as a fall-prevention tool.

## “Gaps” in Current Research

The evidence for medications as risk factors for falls is based on observational studies. To date, RCTs have only rarely collected falls as adverse events [15–17]. The EuGMS Task and Finish group on FRIDs recommends that falls and fall-related injuries should be actively sought as adverse events in RCTs when applying for a license for a new drug to enter the market [52]. In addition, we advocate that older people, including the “oldest old,” are adequately represented in RCTs as they are the primary users of medications [52]. Further research is required to establish the lower limit for blood-pressure reduction using antihypertensives in frailer older adults, after which the harms of falls and other adverse events outweigh the cardiovascular benefits.

Most of the published observational studies on medications as risk factors for falling have several quality issues, including accurate medication and falls ascertainment as well as problems with confounder variables [15–17]. The EuGMS Task and Finish group on FRIDs supports the following items to increase the quality of observational studies further [53, 54].

1. *Population* Studies should be conducted in populations with different characteristics to assess whether different populations have dissimilar medication-related fall risks. The characteristics of the population, such as frailty, should be precisely defined.
2. *Outcome* We support the fall definition and fall ascertainment recommended by The Prevention of Falls Network Europe [55]. A fall should be defined as “an unexpected event in which the participant comes to rest on the ground, floor or lower level,” and falls should be recorded prospectively using daily recording.
3. *Medication*
  - The studied target medication should be precisely defined, preferably using the WHO-recommended anatomical therapeutic chemical (ATC) medication classification system. This will enable clinicians and researchers to harmonize datasets, to compare results for the same medication between different studies, and to summarize evidence through systematic reviews and meta-analyses.
  - As different pharmacological subgroups and individual agents in these subgroups might have different fall-risk-increasing properties, these subgroups and chemical substances should be assessed in future studies.
  - Collecting data on medication use only at baseline in studies makes it likely that medication use by participants may change during follow-up. The aim should be to perform the medication (ATC code, dosage, duration of use) data collection, including over-the-counter medications, at a relevant time interval for the fall to minimize bias in the studies.
  - To date, the Drug Burden Index, which evaluates the cumulative burden of sedatives and anticholinergics, and different measures of anticholinergic burden have been associated with increased fall risk [16]. However, whether dosage is an important factor in falls caused by other medication groups should be evaluated in the future. In addition, investigation of whether fall risks increase after initiation of therapy compared with chronic use is warranted.
  - If falls are evaluated as adverse events in observational studies, then validated criteria such as that of the WHO-Uppsala Monitoring Centre (UMC) and a rigorous assessment process for rating the causality of the event should be used [56, 57].
4. *Confounding factors* Confounders should be carefully chosen. Besides the typical confounders such as comorbidities, indicators of frailty, and other factors related to the fall risk, drug indication, and concurrent medications should be taken into account as possible confounding variables.
5. *Other issues* Combination drugs and therapies and drug–drug interactions and their effect on fall risk have not been actively investigated in studies.

## Conclusion

The EuGMS Task and Finish group on FRIDs advocates more attention to dissemination of knowledge regarding FRIDs, increased insight, and improved practice, including the following:

- encouraging systematic judicious deprescribing by including the following steps: recognizing a possible indication for the medication, searching for a safer alternative, performing shared decision making, and monitoring for symptoms after stopping the FRID.
- disseminating knowledge to healthcare workers, healthcare students, and the older population, which could enable a more active role for older individuals in shared decision making. Medication-related falls should be part of the curriculum for all healthcare students.
- creating a European FRIDs list to be used as a fall-prevention tool.
- increasing knowledge about the risk of falls associated with therapeutic classes and individual medications through well-designed observational studies and RCTs.

The EuGMS Task and Finish group on FRIDs emphasizes that effective knowledge dissemination and improved withdrawal of FRIDs, as a result of that knowledge dissemination, is likely to reduce the number of fall injuries [50, 58].
